# Medicine

**Published:** 1920-09

**Authors:** G. Parker


### Progress of the Medical Sciences.

### MEDICINE.

**Non-nephritic Albuminuria.**—In considering when and how far albumen is to be regarded as a sign of nephritis everyone acknowledges that it may occur without permanent disease of the kidney in passive congestion from direct pressure on the renal veins, and in certain forms of heart and lung disease, in many acute fevers, and in pregnancy, or after cerebral hemorrhage or from the irritation of certain drugs such as turpentine. French enumerates some forty other diseases and morbid states in which it may appear. Recent military experience has shown it in a still wider field, for Maclean in testing 50,000 trained soldiers and 10,000 fresh recruits found albumen in 5.6 per cent., though only in 1 per cent. were there definite signs of disease. Indeed, Collier and others have shown that most healthy persons under violent exercise, such as racing, may pass appreciable amounts. Cold baths often produce a similar result, as do mental strain, terror, and nervousness before examinations.

Herringham suggests that these latter are slight forms of hæmoglobinuria. Then there are the instances of cyclic albuminuria in adolescents, and in cases of lordosis. Probably none of these have any connection with nephritis, from which it is most important to distinguish them.

Mackenzie Wallis[1] insists that in many of these conditions what we really get is chiefly a form of globulin associated with lipoids. If the latter are in great excess the fluid is milky or opalescent, as in pseudochyluria. More often this "euglobulin" does not affect the clearness of the urine, but it may be diagnosed by the fact that it is always precipitated by dilute acetic acid in the cold. Thus when a dilute (33 per cent.) solution of acetic acid is added drop by drop to 10 cc. of urine (which may be also diluted) a white precipitate falls. This was formerly called nucleo-albumen, and is often a source of error, since ordinary boiling tests bring down all forms of protein alike. He finds this lecithin-globulin in most of these non-nephritic albuminurias, sometimes to a large amount; but there is no failure in the work of the kidney, no retention of urea or chlorides, no uræmia, no change in the heart or blood pressure. Hence the importance of a preliminary dilute acetic acid test, confirmed if necessary by a quantitative one by ammonia and ammonium sulphate. In some forms of nephritis, especially parenchymatous, and in eclampsia, there is an excess of pseudoglobulin, but not of euglobulin, containing lecithin, which is alone precipitated by acetic acid in the cold.

If his results are confirmed by experience they will afford an invaluable test for non-nephritic conditions. In a discussion of his paper various cases were brought forward in which large amounts of albumen had appeared at times for years without obvious signs of nephritis, and in some of them the presence of lecithin globulin had been proved. It sometimes occurs too in syphilitics.

A very useful test for renal efficiency was devised by H. Maclean.[2] If a large dose of urea is given to a patient with defective kidneys, it is found that he cannot excrete urea in high concentration, *i.e.* 2 per cent. or over. The method is employed by the Pensions Ministry as follows :—

1. The patient empties the bladder and drinks a solution containing 15 grammes of urea in flavoured water.

2. Water is passed one hour and again two hours after the draught, and the amount of urea measured by the hypobromite method. If great diuresis occurs, say 500 cc. in two hours, a third sample should be taken for analysis. The lower the percentage is below 2 the more inefficient are the kidneys.

F. Kidd[3] points out that albumen in bacilluria is also apt to cause errors. Besides that due to calculi, stricture, enlarged prostate or acute pyelitis, we often meet with it in chronic infections, especially by the Bacillus coli. This urine is generally acid, sometimes containing blood or pus, and may have a fishlike odour. There are frequent calls to pass water, the urine may be clear or show a haze which neither clears up with acetic acid nor liq. potassæ, but gives a shot-silk appearance on stirring. Intermittent pyrexia with rigors is sometimes met with, but the all-important thing is to note the presence of crowds of bacteria and to diagnose the chief types.

Much discussion has arisen on a paper by Prof. Langley, which seemed to show that **electrical stimulation of paralysed muscles** has no beneficial result. If electricity has only a mental influence, or if its curative effects cannot be measured and weighed when correctly used, it had better be laid aside. But there are plenty of instances to the contrary. Thus Sir Thomas Oliver has shown (and anyone can repeat the test for himself) that in electrical treatment of plumbism the lead can be removed from within the patient's body by the current and actually weighed. Now it is well known that Leduc, having divided the sciatic nerves in animals, treated one limb in each case with certain forms of electricity for a long definite period and then killed them and compared the weight of the treated limbs with that of the controls. A large gain of weight was found in the muscles treated by certain currents, and only in those. His results have been confirmed by practice. There seems general agreement that Langley's experiments do not contradict this at all. He too divided the sciatics and carefully weighed the treated and untreated muscles, finding that atrophy was not prevented. But his treatment by condenser currents only was confined to the twenty days after section, *i.e.* to the period of irritation, when stimulation is probably useless and harmful. It is another question what the results would have been had he taken the second period from the twentieth day onwards as in ordinary practice. It is possibly true that nothing will check atrophy in the first fortnight, especially if the muscle is then, as is suggested, in continuous fibrillary contraction.

G. Cooper, in discussing atrophy from disuse[4] points out that apart from actual wounds of the muscle and toxins, it is due to (1) lesions of the lower neuron, (2) immobilisation of the joints as by splints, (3) reflex inhibition of the muscle to give rest to a diseased joint. Nutrition is effected chiefly during contractions by exchanges between the muscle plasma and the lymph (and blood vessels), and there seems to be no difference between contractions produced by normal nerve stimuli and electrical ones as to the metabolism resulting. Hence that type of electrical treatment is most effective which gives the best contractions with the least pain. He advises for this end picking out each muscle with a new form of Faradic battery which gives unvarying interruptions up to 900 per second. Of course, in muscles, which show R.D., galvanism alone produces contractions.

Cumberbatch[5] lays down that besides causing contractions we should stimulate the afferent nerve endings in the skin to influence reflexly the anterior horn cells. The value of this is shown in a case where he obtained a return of voluntary movement after five years' paralysis by using a sinusoidal current for three months, which never produced a single contraction. Macgregor reported a case of plumbism where with precisely similar electrical reactions in both arms the one treated with Faradism recovered even sooner than that to which galvanism was applied. Lewis Jones and others have insisted on the value of Faradism even when the muscles do not react to it. Certainly the coldness, blueness and many trophic changes disappear under it. We require a large number of observations recorded minutely to settle the value of the two methods; meanwhile, after the first three weeks the best plan is to pick out and exercise each muscle by that current which is effective, and also to give Faradism to the limb.

Finally, since fatigue in a muscle is the non-removal of lactic acid, the circulation should be increased before each treatment by hot or whirlpool baths or massage to oxidize and carry it away.

G. Parker.

REFERENCES.

[1] *Proc. Roy. Soc. Med.*, 1920, xiii., Sect. Med., p. 96.
[2] *Brit. J. Exp. Path.*, vol. i., No. 1, p. 53.
[3] Frank Kidd, *Common Infections of the Kidneys*, 1920.
[4] *Proc. Roy. Soc. Med.*, 1920, xiii., Sect. Electro-Therap., p. 33.
[5] *Ibid.*, p. 45.

